# Phlegmasia Cerulea Dolens in a 21-Year-Old Female Patient Following Depo-Provera Injection: A Case Report

**DOI:** 10.7759/cureus.79426

**Published:** 2025-02-21

**Authors:** Shadrack Ansong, Julianne Meany, Chinazor Umerah, Chika Okafor

**Affiliations:** 1 Internal Medicine, Cape Fear Valley Medical Center, Fayetteville, USA; 2 Internal Medicine, Drexel University College of Medicine, Fayetteville, USA

**Keywords:** depo-provera, massive pulmonary embolism, oral contraceptives, phlegmasia cerulea dolens (pcd), surgical thrombectomy

## Abstract

Phlegmasia cerulea dolens (PCD) is a rare and severe form of deep vein thrombosis (DVT) associated with limb ischemia. We present the case of a 21-year-old female patient who presented with lower extremity numbness, pain, and swelling, accompanied by pleuritic chest pain and shortness of breath. She had a recent history of receiving a Depo-Provera shot for birth control. Diagnostic assessments revealed an extensive femoral-to-popliteal DVT and bilateral pulmonary embolisms. The patient underwent a series of interventions, including thrombectomy, catheter-directed thrombolysis, and repeat thrombectomy with venoplasty. She was discharged on anticoagulation therapy and showed favorable outcomes during her hospital stay. This case highlights the potential association between Depo-Provera use and the development of PCD, emphasizing the importance of prompt diagnosis, multidisciplinary management, and follow-up care.

## Introduction

Phlegmasia cerulea dolens (PCD) is a rare and potentially life-threatening condition characterized by extensive venous thrombosis, leading to severe limb ischemia [1]. The term "phlegmasia cerulea dolens" translates to "painful blue edema" in Latin, highlighting the clinical presentation of the condition. It is a limb-threatening complication that requires prompt recognition and intervention to prevent irreversible damage and potential mortality.

PCD is primarily caused by extensive venous thrombosis involving the deep, superficial, and collateral veins [2,3]. It typically affects the lower extremities, although cases involving the upper extremities have also been reported. The condition manifests as acute, severe limb pain, swelling, cyanosis, and compromised arterial blood flow. If left untreated, PCD can progress rapidly, leading to tissue necrosis, gangrene, and, ultimately, limb loss.

While PCD is commonly associated with deep vein thrombosis (DVT) and pulmonary embolism (PE), its occurrence following the administration of the Depo-Provera shot, a widely used contraceptive hormone injection, is a rarely reported phenomenon [1,3,4]. Depo-Provera, containing medroxyprogesterone acetate, is a progestin-only contraceptive method widely used due to its convenience and long-lasting effect [1,2]. Understanding and recognizing the potential association between Depo-Provera use and the development of PCD is crucial to ensuring early diagnosis, appropriate management, and prevention of severe complications.

In this case report, we present a unique case of a 21-year-old female patient with no pertinent past medical history who developed PCD shortly after receiving a Depo-Provera shot.

## Case presentation

A 21-year-old female patient with no significant past medical history or family history of clotting disorders presented to the emergency department (ED) with complaints of lower extremity numbness, pain, and swelling. She reported feeling short of breath for the past week and had been immobile due to back pain, severe lower extremity pain, and edema. The night she presented to the ED, she experienced a sudden onset of shaking chills and severe leg and abdominal pain, more intense than the day prior. The patient denied fever, palpitations, nausea, vomiting, and diarrhea. She had no history of blood clots, recent surgery, recent travel, coronary artery disease, or tobacco use. However, she reported smoking marijuana seven times a week and had received a Depo-Provera shot for birth control approximately a month before her presentation.

In the ED, the patient was found to have reddish-purple discoloration of the entire left leg compared with the right, most obvious proximally. Radial pulses were 2+ on the right side and 2+ on the left side. Dorsalis pedis pulses were 2+ on the right side and 1+ on the left side of the lower extremities. On physical examination, she was in respiratory distress, with bilateral lower extremity tenderness, numbness in the right lower extremity, and tenderness with bluish discoloration of the entire left lower extremity.

Vital signs were significant for a respiratory rate of 22, heart rate of 102, and oxygen saturation of 92% on 4 liters of oxygen via nasal cannula; she had presented with 89% on room air. Initial diagnostic evaluation in the ED, including complete blood count (CBC), complete metabolic panel (CMP), and prothrombin time (PT) with international normalized ratio (INR), is shown in Tables 1, 2, and 3, respectively.

**Table 1:** CBC at the time of admission. CBC: complete blood count; WBC: white blood cell; RBC: red blood cell; MCV: mean corpuscular volume; MCH: mean corpuscular hemoglobin; MCHC: mean corpuscular hemoglobin concentration; RDW-SD: red cell distribution width-standard deviation; MPV: mean platelet volume; RDW-CV: red cell distribution width-coefficient of variation; nRBC: nucleated red blood cell.

Result	Value	Reference Range
WBC	13.8	4.5–12.5 × 10^3^/µL
RBC	4.05	4.20–5.40 × 10^6^/µL
Hemoglobin	12.8	12.0–16.0 g/dL
Hematocrit	37.9	36.0–48.0%
MCV	93.6	81.0–99.0 fL
MCH	31.6	27.0–31.0 pg
MCHC	33.8	31.0–36.0 g/dL
RDWSD	41.0	36.4–46.3 fL
Platelets	279	150–450 × 10^3^/µL
MPV	10.2	7.4–10.4 fL
RDWCV	11.8	11.7–14.4%
nRBC	0.0	<1%
nRBC absolute	0.00	0.00–0.01 × 10^3^/µL

**Table 2:** Complete metabolic panel at the time of admission BUN: blood urea nitrogen; CO₂: carbon dioxide; AST: aspartate aminotransferase; ALT: alanine aminotransferase; eGFR: estimated glomerular filtration rate.

Result	Value	Reference Range
Sodium	137	136–145 mmol/L
Potassium	3.3 (L)	3.4–4.9 mmol/L
Chloride	104	98–107 mmol/L
CO_2_	22	21–32 mmol/L
BUN	5 (L)	7–25 mg/dL
Creatinine	0.70	0.60–1.30 mg/dL
Glucose, random	95	74–109 mg/dL
Calcium	9.4	8.6–10.2 mg/dL
AST	22	13–39 U/L
ALT	21	7–52 U/L
Alkaline phosphatase	60	30–105 U/L
Protein total	8.0	6.4–8.9 g/dL
Albumin	3.7	3.5–5.7 g/dL
Bilirubin total	1.2 (H)	0.3–1.0 mg/dL
eGFR	>60.0	>60.0 mL/min/1.73 m^2^
Anion gap	11	1–11 mmol/L

**Table 3:** PT/INR with aPTT at the time of admission PT: prothrombin time; INR: international normalized ratio; aPTT: activated partial thromboplastin time

Result	Value	Reference Range
Prothrombin time	11.8	9.7-12.4 seconds
INR	1.1	0.9-1.1
aPTT	23.5 (L)	24.3 - 34.6 seconds

Diagnostic assessment

**Figure 1:**
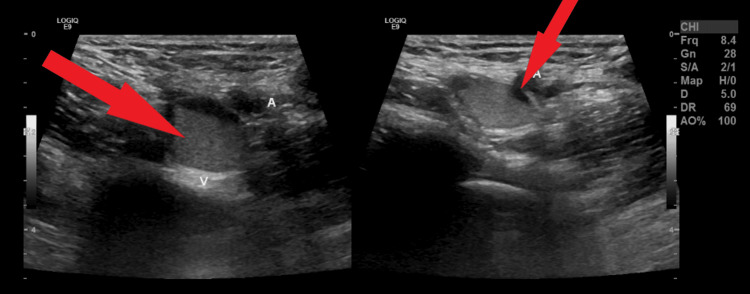
Ultrasound of the left lower extremity showing deep vein thrombosis (DVT), indicated by the red arrows

**Figure 2:**
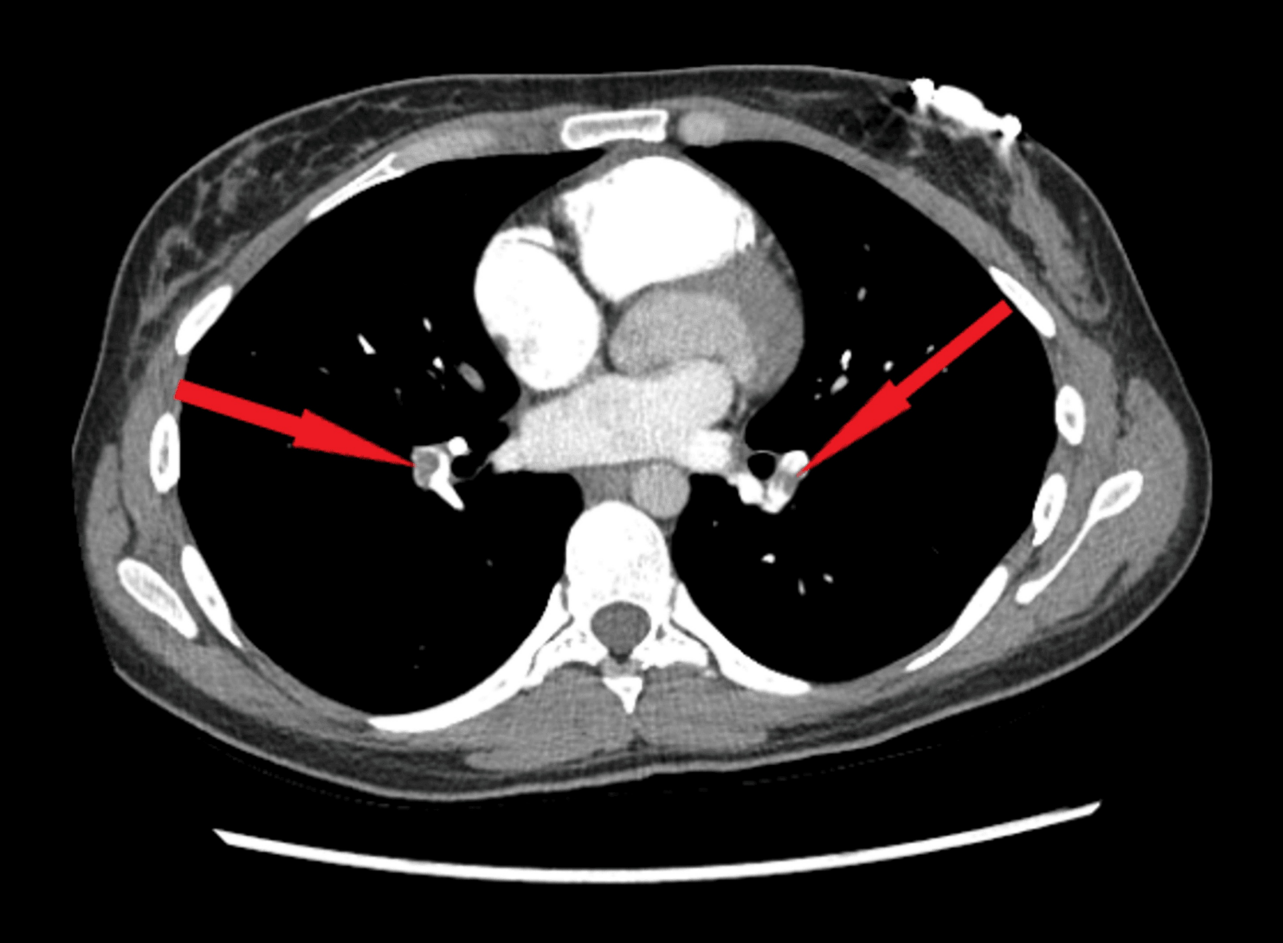
Axial view of CTA of the chest showing bilateral pulmonary embolism, indicated by the red arrows CTA: computed tomography angiography.

The patient's EKG showed no acute ST abnormalities, and troponin levels were within normal limits. A CBC revealed mild leukocytosis without a left shift, as shown in Table 1, while the CMP revealed mild hypokalemia of 3.3, as shown in Table 2. Her PT and INR were within normal limits, except for a slight decrease in the activated partial thromboplastin time (aPTT), as indicated in Table 3. Due to the pain and discoloration of the left lower extremity, a left lower extremity ultrasound with Doppler was performed, revealing an extensive femoral-to-popliteal deep venous thrombosis (DVT), as shown in Figure 1. The patient was presumed to have arterial involvement; therefore, a computed tomography angiography (CTA) of the aorta with bilateral iliofemoral runoff was performed, which showed occlusion of the left below-knee popliteal artery. This was discussed with vascular surgery for a possible intervention. The vascular team considered arterial involvement unlikely but recommended interventional radiology (IR) thrombectomy due to the extensive DVT. CTA of the chest was performed due to pleuritic pain and shortness of breath, revealing left lower lobe segmental and subsegmental pulmonary emboli, as shown in Figure 2. Interventional radiology was consulted, and the patient subsequently underwent a venogram with thrombectomy followed by catheter-directed thrombolysis. She was started on tissue plasminogen activator (tPA) to dissolve the clots and was transferred to the intensive care unit (ICU) for observation.

A repeat CTA of the chest was performed two days later, showing almost complete resolution of the previously noted bilateral PEs. Later that morning, she underwent a repeat venogram, which demonstrated an interval decrease in clot burden. Repeat thrombectomy and venoplasty were performed. The patient experienced hematuria after the procedure and was kept in the ICU for an additional day of observation. Her gross hematuria resolved the following day, and she remained stable. To rule out other causes of hypercoagulability, further testing was conducted, as shown in Table 4, all of which were within normal limits. The patient had no genetic predisposition, as she denied any family history of hypercoagulability.

As part of the initial diagnostic evaluation, blood cultures were drawn. Her C-reactive protein (CRP) level was elevated at 40. A Cepheid test was negative for influenza and COVID-19; both viruses have been reported in the literature to be associated with some cases of PCD. Blood cultures remained negative after 48 hours.

**Table 4:** Diagnostic evaluation for hypercoagulability DNA: deoxyribonucleic acid; ANA: antinuclear antibody.

Investigation	Results	Reference Range
Factor V Leiden mutation (C.1601G>A)	Not detected	Not detected
Protein S free	93%	61–136%
Protein S-functional	65%	63–140%
Protein S total	76%	60–150%
Protein C antigen	105%	60–150%
Protein C functional	110%	72–180%
Factor II DNA analysis (C.*97G>A)	Not detected	Not detected
Antithrombin activity	118%	75–135%
Lupus anticoagulant	Not detected	Not detected
ANA	Negative	Negative
Lupus anticoagulant neutralization dilute phospholipid time (dPT)	36.5 seconds	0.00–46 seconds

Management and outcome

The patient underwent a series of interventions, including a venogram with thrombectomy, catheter-directed thrombolysis, a repeat venogram with thrombectomy and venoplasty, and subsequent administration of tPA. The repeat CTA of the chest revealed almost complete resolution of the bilateral PEs following tPA administration. She experienced a complication of hematuria following the procedure, which eventually resolved without further issues. The patient remained stable during her hospital stay and was discharged on apixaban for continued anticoagulation.

The management approach in this case involved a multidisciplinary team, including interventional radiology, vascular surgery, and the ICU, to provide timely and appropriate interventions for the extensive DVT and associated PEs. The patient's response to treatment was favorable, with an interval decrease in clot burden and resolution of the PEs observed on imaging. The decision to discharge the patient on apixaban aimed to prevent further thrombotic events and ensure continued anticoagulation therapy.

## Discussion

PCD is a rare but severe form of DVT characterized by extensive venous obstruction, leading to significant morbidity and potential limb loss if not treated promptly. The condition manifests as a painful, swollen, and cyanotic limb, primarily affecting the lower extremities, particularly the left leg, due to anatomical predispositions such as the crossing of the left common iliac vein by the right common iliac artery, which can lead to increased venous compression and thrombosis [3,4]. The urgency of addressing PCD cannot be overstated, as delays in intervention can result in irreversible complications, including venous gangrene and systemic shock. The pathophysiology of PCD involves a combination of venous stasis, hypercoagulability, and endothelial dysfunction, which collectively contribute to the formation of obstructive thrombi within the deep venous system [4,5].

Management strategies for PCD have evolved, with a focus on restoring venous patency and preventing complications. Initial treatment typically involves anticoagulation and supportive measures such as leg elevation and fluid resuscitation [5]. However, these measures alone are often insufficient, particularly in cases with impending venous gangrene. In such scenarios, more aggressive interventions, including catheter-directed thrombolysis and mechanical thrombectomy, are recommended to alleviate venous obstruction and restore blood flow. These techniques have shown promising results in improving limb salvage rates and reducing the risk of amputation [6]. The choice of intervention often depends on the severity of the condition and the presence of complications such as compartment syndrome or arterial insufficiency. In cases where significant arterial compromise is present due to elevated compartment pressures, immediate surgical intervention may be necessary to prevent irreversible ischemia [5,7,8]. Additionally, the use of endovascular techniques, such as stenting, has gained traction in recent years, particularly in patients with anatomical anomalies like May-Thurner syndrome, which predispose them to recurrent thrombosis [8]. These approaches aim not only to remove the thrombus but also to address underlying anatomical issues contributing to venous obstruction.

The etiology of PCD can be multifactorial, with several risk factors identified in previous literature. Common risk factors for PCD include immobilization, recent surgery or trauma, malignancy, pregnancy, hormonal therapy (such as estrogen-containing oral contraceptives or hormone replacement therapy), and genetic predisposition (e.g., inherited thrombophilia) [5]. Other factors associated with PCD include sepsis, central venous catheterization, and coexisting comorbidities such as congestive heart failure or chronic kidney disease.

While the association between the Depo-Provera shot and the development of PCD is rare, DVT and, in some situations, PEs have been reported. In a study by Vlieg et al. [9], DVT and PE were found to be three to four times more likely in premenopausal women aged 18 to 50 years using 150 mg of Depo-Provera compared with those using non-hormonal contraceptives. Depo-Provera, a progestin-only contraceptive injection containing medroxyprogesterone acetate (MPA), is widely used due to its convenience and long-lasting contraceptive effect. Some reported side effects include osteoporosis, hypercoagulability, and vaginal yeast infections. The progestin component of Depo-Provera has been implicated in altering coagulation parameters, potentially promoting a prothrombotic state [10].

The exact mechanisms through which Depo-Provera may contribute to the development of PCD remain speculative and require further investigation. It has been postulated that the progestin component could lead to changes in the coagulation cascade, endothelial function, and venous stasis, thereby increasing the risk of venous thrombosis. However, the available evidence is limited, and the association between Depo-Provera and PCD requires more comprehensive studies to establish a causal relationship [3].

This patient received 150 mg of Depo-Provera four weeks before her presentation. As stated above, several other factors can enhance hypercoagulability, including infections, genetic predisposition, and environmental exposures. On presentation, sepsis secondary to skin abscess, cellulitis, and gangrene was high on the differential. Imaging helped eliminate infectious causes, and blood cultures remained negative at 24 and 48 hours. Due to the timely involvement of a multidisciplinary team, including interventional radiology and vascular surgery, a prompt decision was made to proceed with thrombectomy and venoplasty, thereby preventing the development of gangrene, one of the reported complications of PCD, and limb ischemia [8].

Considering the potential association between Depo-Provera and PCD, healthcare providers should be vigilant in assessing patients for risk factors and monitoring for any signs or symptoms of thromboembolic events. A thorough evaluation of the patient's medical history, including medication use and risk factors for thrombosis, should be performed. If PCD is suspected, prompt diagnostic assessments, such as ultrasound imaging, should be pursued to confirm the diagnosis and guide appropriate management strategies.

## Conclusions

In conclusion, while the association between Depo-Provera and the development of PCD is rare, it is important to consider this potential complication, particularly in young individuals without significant medical history. Although the exact mechanisms remain unclear, documented cases have linked the Depo-Provera shot to PCD. Further research is needed to elucidate the underlying pathophysiology and establish a definitive causal relationship. Healthcare providers should remain vigilant in assessing and managing patients receiving Depo-Provera to ensure early recognition and timely intervention in cases of suspected PCD.
